# The Effects of Elexacaftor, Tezacaftor, and Ivacaftor (ETI) on Blood Glucose in Patients With Cystic Fibrosis: A Systematic Review

**DOI:** 10.7759/cureus.41697

**Published:** 2023-07-11

**Authors:** Marcelo Salazar-Barragan, Daniel R Taub

**Affiliations:** 1 Biology, Southwestern University, Georgetown, TX, USA

**Keywords:** continuous glucose monitoring (cgm), glycated hemoglobin (hba1c), ogtt, blood glucose control, cystic fibrosis (cf), cystic fibrosis triple therapy, cystic fibrosis transmembrane conductance regulator, cystic fibrosis related diabetes

## Abstract

Cystic fibrosis (CF) is an autosomal recessive genetic disorder resulting from defects in the cystic fibrosis transmembrane conductance regulator (CFTR) protein, which in turn results in a multi-systemic disorder. There are numerous known CF alleles associated with different mutations of the CFTR gene, with the most common CF allele being a three-base-pair deletion known as ΔF508. One common manifestation of CF is glycemic dysregulation associated with decreased insulin secretion, often progressing into a distinct form of diabetes known as cystic fibrosis-related diabetes (CFRD). In the past decade, a class of drugs known as CFTR modulators has entered clinical practice. These drugs interact with the CFTR protein to restore its function, with different modulators (or combinations of modulators) suitable for patients with different CFTR mutations. Previous research has established that the modulator ivacaftor is effective in decreasing blood glucose and sometimes resolving CFRD in patients with certain CFTR mutations (class III mutations). However, early modulator therapies for individuals with the common ΔF508 mutation (e.g., a combination of the modulators lumacaftor and ivacaftor) have largely proven ineffective in improving glucose regulation. More recently, a combination therapy of three modulators, namely elexacaftor, tezacaftor, and ivacaftor (ETI), has entered clinical practice for patients with the ΔF508 mutation. However, it is not clear whether this therapy is effective in treating dysglycemia.

We searched for studies of any design that examined the effects of ETI on measures of blood glucose. All available studies were observational studies comparing patients before and after initiating ETI therapy. Measures of daily-life blood glucose (those obtained with continuous glucose monitoring systems or by measuring glycated hemoglobin (HbA1c)) and post-prandial glucose spikes from oral glucose tolerance tests showed significant improvements in at least some studies. The majority of studies showed significant improvements from pre- to post-ETI in one or more blood glucose measures. While the interpretation of this evidence is complicated by the lack of randomized controlled trials, it appears that ETI therapy is associated with improved glucose regulation for at least some patients with the ΔF508 mutation.

## Introduction and background

Cystic fibrosis (CF) is an autosomal recessive inherited disorder that affects more than 100,000 people worldwide [1]. It is caused by mutations in the cystic fibrosis transmembrane regulator conductance (CFTR) gene, which encodes a transport protein involved in bicarbonate and chloride secretion found abundantly on the surface of epithelial cells lining the lungs, intestines, and exocrine pancreas [2]. 

As of April 7, 2023, the CFTR2 database [3] lists 719 distinct CF-causing genetic variants of the CFTR gene, along with 49 variants of varying expressivity. Mutations in the CFTR gene can be classified into six classes according to their molecular deficits [4]. Classes I to III result in the most severe manifestations of the disease due to a complete lack of function of the CFTR protein. In contrast, proteins associated with classes IV to VI retain some degree of proper CFTR functioning; so individuals with these mutations have better clinical presentations [5]. The most common CF mutation is the ΔF508 mutation, which is a class II mutation with a deletion of three bases that code for a phenylalanine residue in the protein, leading to improper folding of the protein. Worldwide, approximately two-thirds of all CF alleles are ΔF508 [6]. 

Common clinical manifestations of CF include glucose regulation abnormalities, which develop in approximately 50% of CF adults over 30 into a distinct form of diabetes known as cystic fibrosis-related diabetes (CFRD) [7]. It is typically diagnosed by a 2-hour oral glucose tolerance test (OGTT) plasma glucose ≥200 mg/dl or fasting plasma glucose ≥ 126 mg/dl [8]. The pathogenesis of CFRD is complex and not entirely understood, but may involve defects in the CFTR protein interfering with insulin production and secretion [9,10], as well as scarring of the pancreas [9,11]. The prevalence of CFRD in individuals increases with age, with one study on CFRD patients in Europe finding that 0.8% of children and 9.7% of adolescents with CF had CFRD, with the prevalence increasing to about 32% in young adults [12]. 

CFTR modulators and their effects on blood glucose

Recent years have seen the adoption of a new class of drugs for CF called modulators, which directly interact with defective CFTR proteins to improve their functioning. The most common CFTR modulator molecules either act as a corrector, aiding the CFTR protein in folding into its proper tertiary structure, or as a potentiator, improving the ability of the CFTR protein to remain open to transporting chloride at the cellular membrane [13]. The first CFTR modulator was a potentiator, ivacaftor (Kalydeco), approved by the US FDA in 2012 for use by individuals with certain class III CFTR mutations that have a defect that inhibits adenosine triphosphate (ATP)-dependent channel gating [14]. For individuals with the ΔF508 mutation, the first two modulator therapies were Orkambi (lumacaftor-ivacaftor) and Symdeko (tezacaftor-ivacaftor), which were FDA-approved in 2015 and 2018, respectively [15,16]. The ΔF508 mutation is a class II mutation, leading to a protein that is misfolded and poorly trafficked to the cellular membrane. The ΔF508 protein is also deficient in transport activity even when it is properly folded and present at the cell membrane. Both of these modulator therapies for ΔF508, therefore combine a corrector (lumacaftor or tezacaftor) with the potentiator ivacaftor [16].

Ivacaftor therapy has been shown in several studies to improve the glycemic status and resolve CFRD for many individuals with class III CFTR mutations, while studies on the dual modulator therapy lumacaftor-ivacaftor for those with the ΔF508 mutation have largely suggested that it is ineffective in this regard [16,17]. We are not aware of any studies on the effectiveness of the tezacaftor-ivacaftor combination in improving glycemic status for individuals with ΔF508 [17]. 

Trikafta

Trikafta is a newer CFTR combination drug therapy that was FDA approved in 2019 for individuals aged 12 years and older with at least one copy of the ΔF508 mutation (FDA, 2019). In Europe, a combination of the same drugs goes by the name Kaftrio and was authorized for use in the E.U. in 2020 [18]. Trikafta consists of elexacaftor, tezacaftor, and ivacaftor (ETI). Elexacaftor and tezacaftor are two corrector molecules, and ivacaftor is a potentiator. Trikafta has been shown to be superior to dual modulator therapies for a variety of CF-related measures, including forced expiratory volume (FEV1), sweat chloride levels, BMI, and the number of pulmonary exacerbations [19,20]. However, the effects of ETI on patient glycemic status and CFRD have received less study, and to our knowledge, no previous review has focused on the effects of ETI on blood glucose levels. 

## Review

Search and data extraction 

We searched for studies on human subjects of any age taking ETI either in a randomized controlled trial (against either placebo or another modulator therapy) or in an observational study. Outcomes of interest were measurements of blood glucose status, including fasting blood glucose, the area under the curve (AUC) from OGTT, glycated hemoglobin (HbA1c), and data from continuous glucose monitors (CGM) on average blood glucose, time in target range, or time in hypoglycemia. We did not include data from random blood glucose measurements, i.e., single measurements taken without regard to recent food consumption. 

Searches were conducted in PubMed on 13 April 2023, searching all fields for the terms: (elexacaftor OR elexacaftor/tezacaftor/ivacaftor OR ETI OR trikafta) AND (glycemi* OR diabet* OR CFRD OR A1C OR glucose), and filtering for papers in the English language. Additional searches in Google Scholar and in the reference sections of papers yielded no additional studies with relevant data. 

**Figure 1:**
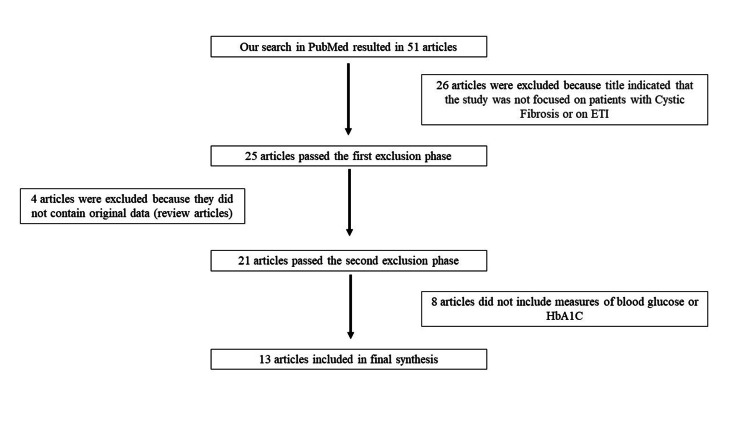
Flow chart or process of identification of papers for review ETI: Elexacaftor, tezacaftor, and ivacaftor; HbA1C: Glycated hemoglobin

A review of the literature proceeded as outlined in Figure 1. Both authors assessed papers independently at each step, and any disagreements were resolved by discussion. The final list of papers was once again assessed by each author for relevant data, and any disagreements that arose were resolved via discussion. 

Characteristics of the studies identified

All studies identified were observational studies comparing patients prior to initiating ETI therapy and post-ETI initiation. A lack of randomized controlled trials addressing this topic has been noted previously [16]. Most studies we identified (10 of 13) performed their follow-up (post-ETI) either exclusively within one year after beginning ETI therapy or with a median value among patients of less than 12 months since beginning ETI therapy (Table 1).

**Table 1:** Characteristics of studies on the effects of Trikafta (ETI) on blood glucose in patients with CF ETI: Elexacaftor, tezacaftor, and ivacaftor; CF: Cystic fibrosis; CFRD: Cystic fibrosis-related diabetes; CGM: Continuous glucose monitor; OGTT: Oral glucose tolerance test; FEV1: Forced expiratory volume in 1 second

Authors and year of study	Participants' CFRD status	Percentage of F508 homozygotes in study	Study design and country of study	Participant inclusion and exclusion criteria	Timeframe of pre and post-ETI glucose data
Benninger et al., 2021 [21]	Not specified	100%	Retrospective (single center), United States	Inclusion: Adult CF patients, who previously had bilateral lung transplantation, taking ETI, homozygous for F508; Exclusion: Not specified	Pre-ETI: Within 1 year prior to beginning ETI; Post-ETI: Weekly after ETI
Causer et al., 2022 [22]	CFRD	100%	Case series (single center), England	Inclusion: CF patients homozygous for F508, consenting to phase III ETI clinical trial; Exclusion: Not specified	Pre-ETI: Within 14 days of ETI start date; Post-ETI: 14 days immediately after ETI therapy
Chan et al., 2022 [23]	Mix	40%	Prospective (multi-center), United States	Inclusion: ≥6 years old with CF, taking ETI for at least 8 weeks prior to the study; Exclusion: Type I or type II diabetes, undergone organ transplantation, pregnancy, individuals taking insulin or glucose homeostasis medications, antibiotic use within 4 weeks of starting the study, hospitalization or steroid usage within 8 weeks of starting the study	Pre-ETI: Within 1 year prior to starting ETI; Post-ETI: Median 10.5 months after starting ETI
Crow et al., 2022 [24]	CFRD	Not specified	Retrospective (single center), United States	Inclusion: Adult patients, diagnosis of CFRD, utilization of Dexcom G6 sensor, have been taking ETI for ≥6 months; Exclusion: Had no CGM data for at least 7 days within 3 months before starting ETI and within 6 months after starting ETI, hospitalizations or infections during the study period	Pre-ETI: 3 months before starting ETI; Post-ETI: Both 3 months and 6 months after starting ETI
Korten et al., 2022 [25]	Mix	56%	Prospective (single center), Switzerland	Inclusion: Taking ETI, pediatric population (≥12 years old), ≥1 copy of F508; Exclusion: CFRD patients	Pre-ETI: Median 3 days before initiation of ETI (OGTT), Between day 1 and day 3 before ETI initiation (CGM); Post-ETI: Between day 2 and day 6 post-ETI (CGM) and a median 26 days after ETI initiation (OGTT)
Petersen et al., 2022 [26]	CFRD & non-CFRD data reported separately	58%	Retrospective (single center), United States	Inclusion: Taking ETI, adult patients; Exclusion: Pregnancy within 1 year before starting ETI or during ETI initiation, lung transplant recipient within 1 year before or any time after starting ETI, not following dosage of ETI, limited data during the 3-month period after starting ETI, clinical trial patient	Pre-ETI: Closest to date before starting ETI but not more than 12 months prior to starting ETI; Post-ETI: Most recent date after starting ETI
Piona et al., 2022 [27]	Mix	Not specified	Prospective (multi-center study), Italy	Inclusion: ≥ 6 years old with CF, ≥1 F508 mutation and taking ETI, FEV1 ˂ 40%, Exclusion: Medications affecting glucose homeostasis within 6 weeks prior to starting the study, pulmonary exacerbation within 6 weeks prior to the study, presence of liver/kidney disease, lung/liver transplant recipient	Pre-ETI: Within 1 to 12 weeks of starting ETI; Post-ETI: 12 to 18 months after starting ETI
Ramos et al., 2022 [28]	Mix	Not specified	Retrospective (multicenter), United States and Canada	Inclusion: CF lung transplant recipients who were prescribed ETI after lung transplantation; Exclusion: Not specified	Pre-ETI: Not Specified; Post-ETI: Median 164 days
Scully et al., 2022 [29]	CFRD & non-CFRD data reported separately	CFRD patients: 59%; Non-CFRD patients: 29%	Prospective (single center), United States	Inclusion: ≥1 F508 mutation, plan on beginning ETI therapy; Exclusion: Pregnancy, ages <18 or >70	Pre-ETI: Within 3 months before starting ETI; Post-ETI: Within 12 months after starting ETI
Stekolchik et al., 2022 [30]	CFRD	0%	Case study, United States	Inclusion: Not specified; Exclusion: Not specified	Pre-ETI: Within 10 months of initiating ETI therapy; Post-ETI: Within 10 months after initiating ETI
Granados et al., 2023 [31]	Mix	50%	Secondary analysis (multicenter), United States	Inclusion: >6 years of age, naive to ETI at the beginning of the study; Exclusion: Pancreatic insufficiency, type I or type II diabetes, organ transplant, pregnant, taking medications that affect glucose metabolism, initiation of antibiotics within 4 weeks of study start date, hospitalization or steroid usage within 8 weeks of study date	Pre-ETI: Within 1 year of ETI start date; Post-ETI: Within 1 year after starting ETI
Park et al., 2023 [32]	CFRD	86%	Case series (single center), England	Inclusion: Pediatric population >12 years of age, taking ETI, diagnosed with CFRD; Exclusion: Not specified	Pre-ETI: 5 days prior to ETI initiation; Post-ETI: 14 days and 5 to 13 months after ETI initiation
Steinack et al., 2023 [33]	MIx	64%	Retrospective (single center), Switzerland	Inclusion: Eligible for ETI, ≥18 years, OGTT data available; Exclusion: Taking insulin or hypoglycemia medications throughout 3 months prior to ETI initiation	Pre-ETI: Median 309 days prior to ETI initiation; Post-ETI: Median 184 days after starting ETI

Six of the studies reported data from a patient population identified as exclusively CFRD, with two of these studies also reporting data from a patient population identified as non-CFRD. The other seven studies either presented data from a combined CFRD and non-CFRD patient population or did not specify patient CFRD status (as seen in Table 1). The most common form of blood glucose measurement was HbA1c, with data reported by nine studies (Table 2). Data from continuous glucose monitor (CGM) devices were reported in six of the studies; results of the OGTT were reported in four studies; and several studies reported multiple measures of blood glucose. Patient sample sizes were generally small, with a median sample size of 11 across all measures from all studies (Table 2). Two studies [22,30] reported glycemic measures for only a single patient. 

**Table 2:** Summary of results from studies on the effects of Trikafta (ETI) on blood glucose *Data reported as means, ⁺Data reported as medians (as per original studies) ETI: Elexacaftor, tezacaftor, and ivacaftor; NS: Not significant; NA: Not available; CFRD: Cystic fibrosis-related diabetes; CGM: Continuous glucose monitor; iAUC: Incremental area under the curve; AUC: Area under the curve; HbA1c: Glycosylated hemoglobin

Authors and year of study	Sample subset	N	Measurement	Units	Pre-ETI value	Post-ETI value	P-value
Benninger et al. (2021) [21]	-	9	Fasting glucose	*mg/dL	124	95.7	<0.02
Causer et al. (2022) [22]	-	1	CGM: Avg. glucose	mg/dL	106	103	-
Chan et al. (2022) [23]	-	9	CGM: Avg. glucose	⁺mg/dL	109	87	0.10
	-	9	CGM: Time in target	⁺%	95	85	0.08
	-	20	Glucose iAUC	⁺mg/dL	6317	6599	0.87
	-	20	Fasting glucose	⁺mg/dL	92	91	0.70
	-	15	HbA1c	⁺%	5.5	5.4	0.003
Crow et al. (2022) [24]	3 months post-ETI	11	CGM: Avg. glucose	⁺mg/dL	153.6	147.4	0.24
	6 months post-ETI	11	CGM: Avg. glucose	⁺mg/dL	153.6	143	0.52
	3 months post-ETI	11	CGM: Time in target	⁺%	76.1	75	0.12
	6 months post-ETI	11	CGM: Time in target	⁺%	76.1	73	0.32
	3 months post-ETI	5	HbA1c	⁺%	7.1	7.2	>0.99
	6 months post-ETI	5	HbA1c	⁺%	7.1	6.8	0.88
Korten et al. (2022) [25]	-	11	CGM: Avg. glucose	⁺mg/dL	120	119	0.60
	-	11	CGM: Time in target	⁺%	81.11	81.13	0.50
	-	15	AUC	⁺(mmol L^-1^ min^-1^)	1384	1262	0.008
	-	15	Fasting glucose	⁺mg/dL	93	92	0.20
	-	16	HbA1c	⁺%	5.7	5.6	0.60
Petersen et al. (2022) [26]	CFRD patients	46	Rate of change in HbA1c	*%/year	n.p.	-0.17 %	0.25
	Non-CFRD patients	57	Rate of change in HbA1c	*%/year	n.p.	-0.16%	<0.005
Piona et al. (2022) [27]	-	5	HbA1c	%	n.p.	n.p.	0.04
Ramos et al. (2022) [28]	-	44	HbA1c	⁺%	6.2	5.8	<0.05
Scully et al. (2022) [29]	CFRD patients	14	CGM: Avg. glucose	*mg/dL	162	144	0.033
	Non-CFRD patients	9	CGM: Avg. glucose	*mg/dL	96	94	0.314
	CFRD patients	14	CGM: Time in target	*%	63.6	73.5	0.011
	Non-CFRD patients	9	CGM: Time in target	*%	73.4	77.6	0.953
Stekolchik et al. (2022) [30]	-	1	HbA1c	%	7.8	8.7	-
Granados et al. (2023) [31]	-	8	Glucose iAUC	⁺mg/dL	6669	5761	0.23
Park et al. (2023) [32]	14 days post-ETI	5	CGM: Avg. glucose	⁺mg/dL	112	108	NA
	5 to 13 months post-ETI	5	CGM: Avg. glucose	⁺mg/dL	112	117	NS
	14 days post-ETI	5	CGM: Time in target	⁺%	95	94.5	NA
	5 to 13 months post-ETI	5	CGM: Time in target	⁺%	95	93.5	NS
	5-13 months post-ETI	4	HbA1c	⁺%	5.9	5.8	NA
Steinack et al. (2023) [33]	-	33	AUC	*(mmol L^-1^ min^-1^)	1541	1250	0.042
	-	33	Fasting glucose	*mg/dL	90	88	0.459
	-	31	HbA1c	*%	5.5	5.39	0.039

Effects of ETI on blood glucose measures

Overall, these 13 studies suggest that ETI may improve at least some measures of blood glucose control for many CF patients. Eight out of the 11 studies with a patient sample size greater than one reported a statistically significant improvement in at least one blood glucose measure (Table 2). The studies that did not find any statistically significant improvements [24,31,32] were all ones with small sample sizes, ranging from 4 to 11 patients for each measure. Across all studies, four of six (67%) comparisons with a patient sample >20 showed a statistically significant improvement in blood glucose measures pre- and post-ETI, compared to only six of 28 (21%) comparisons with a sample size ≤ 20, suggesting that small sample sizes may have been limiting the power of studies to detect the effects of ETI (Table 2).

Each of the glucose variables we examined related to hyperglycemia (CGM average glucose, CGM time in target range, fasting blood glucose, OGTT AUC/incremental area under the curve (iAUC), and HbA1c) showed a statistically significant improvement in blood glucose levels after ETI initiation in at least one study in our review (Table 2). Data collected by CGM appear to show significant improvements in glucose regulation less frequently than in studies using other methodologies. It is not clear why this might be the case, as CGM glucose averages are generally very well correlated with HbA1c measurements for CF populations [34]. Chan et al. [23] suggested that ETI might affect HbA1c measurements by affecting hemoglobin dynamics rather than blood glucose. Unfortunately, no studies included in our review performed both CGM and HbA1c measurements on an identical set of patients, making direct comparisons of these values impossible. It might therefore be that CGM measures were less likely to show a beneficial effect of ETI than other blood glucose measures due to unrelated differences among studies in patient populations or aspects of study design. 

While there have been reports of hypoglycemia associated with CFTR modulator therapy [35], no study showed a significant increase in measures of hypoglycemia, although Chan et al. [23] did closely approach significance for time spent with CGM-measured glucose <70 mg/dl (Table 3).

**Table 3:** Percentage of time in hypoglycemia from CGM studies on the effects of Trikafta (ETI) *Data reported as means, ⁺Data reported as medians (as per original studies) ETI: Elexacaftor, tezacaftor, and ivacaftor; NA: Not available; NS: Not significant; CFRD: Cystic fibrosis-related diabetes, CGM: Continuous glucose monitor

Authors and year of study	Sample subset	N	Plasma glucose range	Pre-ETI (% time)	Post-ETI (% time)	P-value
Chan et al. (2022) [23]	-	9	<70 mg/dL	2.2	14.9	0.06
	-	9	< 54 mg/dL	0.3	1.3	0.80
Crow et al. (2022) [24]	3 months post-ETI	11	< 70 mg/dL	0.5	0.9	0.41
	6 months post-ETI	11	< 70 mg/dL	0.5	0.8	0.76
	3 months post-ETI	11	< 54 mg/dL	0.19	0.14	0.32
	6 months post-ETI	11	< 54 mg/dL	0.19	0.13	0.83
Korten et al. (2022) [25]	-	11	< 60 mg/dL	0	0	0.40
	-	11	< 48 mg/dL	0	0	-
Scully et al. (2022) [29]	CFRD patients	14	< 70 mg/dL	2.9	3.4	0.660
	Non-CFRD patients	9	< 70 mg/dL	18.2	17.8	0.235
	CFRD patients	14	< 54 mg/dL	0.3	0.5	0.228
	Non-CFRD patients	9	< 54 mg/dL	6.8	2.5	0.550
Park et al. (2023) [32]	14 days post-ETI	5	< 54 mg/dL	1.5	6.0	NA
	5 to 13 months post-ETI	5	< 54 mg/dL	1.5	2.0	NS

**Figure 2:**
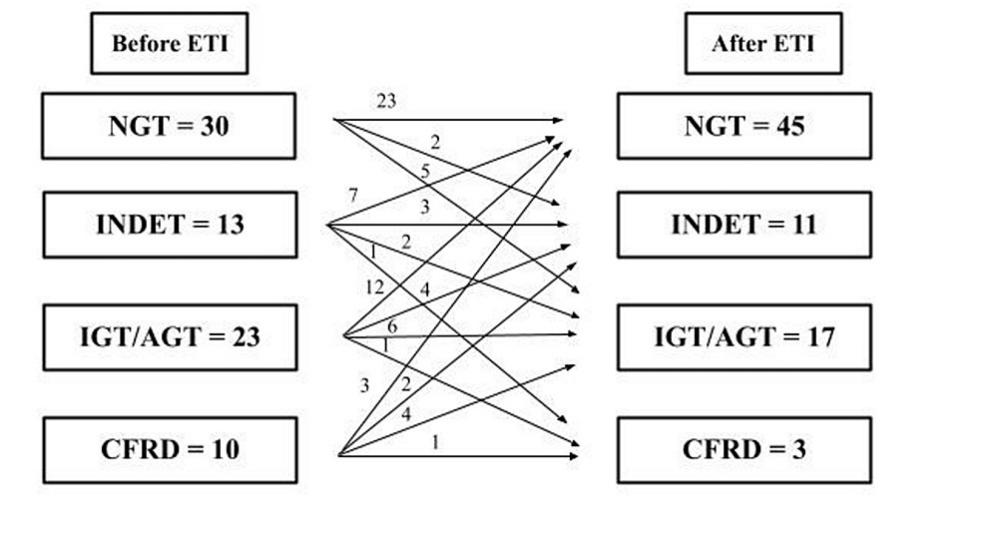
Patient transitions in glycemic status on OGTTs before and after initiating ETI therapy ETI: Elexacaftor, tezacaftor, and ivacaftor; NGT: Normal glucose tolerance; INDET: Indeterminate glucose tolerance; IGT: Impaired glucose tolerance; AGT: Abnormal glucose tolerance; CFRD: Cystic fibrosis-related diabetes; OGTT: Oral glucose tolerance test Data for this figure created by the authors have been combined from [24,27,32,34]. The terms and categorizations are those of the authors of each paper.

While the overall effect of initiating ETI treatment appears to have been an improvement in glycemic status, there appears to be meaningful variation in response among individual patients. Several studies in our review reported transitions for individual patients between categories of glycemic control (i.e., normal glucose tolerance, abnormal glucose tolerance, CFRD) after initiating ETI therapy (Figure 2). While a substantial number of patients transitioned to a category indicating improved glucose tolerance (e.g., a transition from CFRD to impaired glucose tolerance or from impaired to normal glucose tolerance), a few patients exhibited changes in the other direction toward less effective glycemic control (Figure 2). Similarly, Park et al. [32] noted that six of the seven patients they studied were able to decrease or entirely eliminate the use of insulin following initiation of ETI, but one patient with an extremely high HbA1c at baseline saw her HbA1c value increase dramatically after beginning ETI therapy and slightly increased her insulin dosage.

It is not clear whether the effects of ETI may differ between patients based on prior CFRD diagnosis. Two studies [26,29] reported data separately for CFRD and non-CFRD individuals. Scully et al. [29] found that average glucose, time spent in hyperglycemia (plasma glucose > 200 mg/dl), and time spent in the target range with CGM improved in CFRD, while only time spent in hyperglycemia improved significantly in non-CFRD patients. In contrast, Petersen et al. [26] found that HbA1c decreased significantly in non-CFRD patients but not in CFRD patients following the initiation of ETI therapy. However, the mean change in HbA1c was actually slightly greater for patients with CFRD than non-CFRD patients (-0.17% yr-1 vs -0.16% yr-1, respectively) in their study. There were also much broader confidence intervals for the mean HbA1c decrease for patients with CFRD than non-CFRD patients, suggesting possibly greater variability in response for the CFRD population.

Possible mechanisms for the effect of ETI on blood glucose

The mechanisms by which ETI therapy might affect blood glucose are not well understood. If CFTR is expressed in islet β cells, amelioration of molecular defects in the protein might directly impact insulin secretion [29]. However, studies are inconsistent as to whether CFTR is expressed in islet cells [16]. The principal site of CFTR expression in the pancreas is in the ductal epithelium, and there are suggestions that CFTR might affect islet function by paracrine mechanisms [36], providing a mechanism by which amelioration of molecular defects in CFTR might affect insulin secretion. Alternatively, the effects of CFTR correction and potentiation on insulin and glucose homeostasis might be indirectly mediated by effects on incretin secretion or by improvements in inflammation and/or patient nutritional status [16,26,29,33]. 

Limitations of studies

There are a number of limitations and potential biases in the studies included in this review. All of the studies were observational studies performed on patients receiving ETI without a comparator group or blinding of participants. The results of these studies are therefore subject to potential biases, including those arising from placebo effects and those arising from changes over time due to the natural history of CF in these patients [37]. An additional issue common to many of the studies is that they typically had small sample sizes, limiting their power to detect possible effects of the therapy. Steinack et al. [33] performed power calculations for their OGTT study of ETI effects, concluding that a sample size of 30 patients was necessary to have appropriate power to detect an effect of the therapy. Besides the data from Steinack et al. [33], only three of the observations included in Table 2 have a comparable sample size of 30 or more.

The magnitude and consistency of the observed effects of ETI on blood glucose may also have been affected by other pharmaceuticals that the subjects were taking or had taken. At least seven of the studies included some patients who were taking other modulators prior to beginning ETI [23-26,29,31,33]. The effect on blood glucose regulation of newly beginning modulator therapy with ETI would plausibly be greater than the effect of switching from another modulator to ETI. Crow et al. [24] reported that time spent with glucose > 120 and > 140 mg/dl were both significantly improved with ETI for patients naive to modulator therapy, while this improvement was not significant for their study population as a whole. If this is generally the case, then the studies in this review (all but two of which either include a mixed population of patients with and without prior modulator experience or do not specify prior use status) are likely to underestimate the effects of ETI compared to no modulator. In addition, in many of the studies, some patients were insulin users. The authors of several of the studies mentioned that some of their subjects either decreased their dosages or stopped using insulin completely after initiating ETI therapy [21,24,28,32,33]. The reported mean (or median) effects of ETI on blood glucose in these studies may therefore underestimate the effects that would be seen absent from these changes in insulin use. 

## Conclusions

Trikafta or ETI, has beneficial effects on blood glucose in studies comparing patients with CF before and following the initiation of ETI therapy. These studies measured average daily-life blood glucose (such as with CGM and HbA1c) and post-prandial glucose spikes via OGTT. Trikafta appears to have more consistent beneficial effects on glucose levels compared to lumacaftor-ivacaftor therapy, the prior modulator treatment for patients with the most common CF mutation in the CFTR gene, ΔF508. There does appear to be some variation among individuals, with a few having been observed to have worsening glycemic status following the initiation of ETI. While there have been concerns that CFTR modulator therapies may lead to increased incidents of hypoglycemia, this has not been the case in the studies that have been performed to date.
